# Institutional Notes and News

**Published:** 1911-04-08

**Authors:** 


### Institutional Notes and News.

**New Wing Opened at Liverpool.**

The opening of the new out-patient department at the Liverpool Royal Infirmary by Lord Sefton was made unusually important by the speeches that were delivered by Mr. Edmund Owen, Sir Henry Burdett, and the Lord Mayor. Mr. Owen warned the Infirmary authorities against putting the equipment of the costly new out-patient department into competition with the medical men of the neighbourhood. He added that he was not satisfied that the almoner's department was large enough, and urged the abolition of subscribers' letters. He said that the out-patient department should be made as far as possible a consultation department for medical men in the neighbourhood, but that this would never be done if the department began to steal the private practitioner's cases. The Lord Mayor recalled the fact that for a century and a half the Corporation had supported the Infirmary. It is now hoped that the ratepayers will sanction a gift towards the new department. The Lord Mayor also remarked on the willingness to co-operate which was shown by the hospitals in Liverpool. Sir Henry Burdett commended the new unit which enabled the classification of patients to be carried out by means of a casualty ward and a consultative department, so that, after first treatment, patients can be passed on to private practitioners, to provident dispensaries, or to the Poor Law. Sir Henry suggested that, as in America, the patient should have to prove his poverty, and gave high praise to Mr. Ralph Brocklebank, Chairman of the Committee. Mr. Moore, the superintendent, and Mr. J. Francis Doyle, the architect, were cordially thanked for their services.

**Worcester General Infirmary.**

The Bishop of Worcester, as chairman at the annual meeting of Worcester General Infirmary, remarked that there were two points dealing with the position of the Institution, on which he hoped there would be some discussion. First, the building of the new wing, which would make the coming year a very important one. Secondly, the general financial position, which had already occupied the anxious attention of the Executive Committee. The Mayor announced that more than £1,000 had been already received for the proposed Memorial to King Edward—and he felt confident the other £1,000 would be raised in due course. In answer to a question from one of the governors, Colonel Stallard announced that the accounts are now being kept on the system advocated by Sir Henry Burdett. Mr. Gostling, the Senior Hon. Surgeon, replying for the surgical staff, said that 180 abdominal operations had been performed during the year, and the staff could not hope to reduce the expenditure. The Bishop, in returning thanks, expressed his satisfaction on hearing the Mayor's statement with regard to the Memorial Fund. As to the financial position, he suggested that the committee should be asked to consider the whole subject, and report to the Governors at the next annual meeting. The committee had already done their very best to assure the public mind that they were businesslike, and that feeling would be further inculcated if a definite report was submitted to the Governors. He did not want the outgoings to be brought down to the income, but the income to be brought up to the outgoings. There was one method which would be very unpopular, but which he, personally, would take his life in his hands and venture upon. That was, that those who were receiving treatment and could afford to contribute to the funds should do so. He instanced what was done at Guy's Hospital, where they were able to re-open wards which had been closed, by adopting a sliding scale of contributions from the patients. Some people might say that would abolish the idea of charity, but to his mind the best charity was the discriminating kind, and charity was not the less charitable because it helped people to help themselves.

**Birmingham Eye Hospital's Report.**

In moving the adoption of the annual report of the Birmingham and Midland Eye Hospital last week, the Bishop of Birmingham said it was impossible to look at the results of the medical inspection of school children without perceiving the widespread need there was for the care of the eyes. They needed to do their best to afford the most efficient assistance to those who had not the means to pay large fees for the care of their eyes when they consented to apply for remedies. He was glad to see an increase in the work of the hospital under all heads, including that of paying-patients. And here he should like to say that he for one felt strongly in regard to the hospitals that we needed someone of organising power and statesman's faculty for seeing how we should on a very large scale approach the subject of providing such medical treatment as people got at the hospitals on a basis intermediate between that of charitable institutions and that of expensive nursing homes. He was quite sure that we wanted something between the payment at a high fee for surgical and medical attendance possible only to the comparatively wealthy and the merely gratuitous charitable treatment, and we wanted it on a large scale. The report, which was presented by Mr. Walter Wilkinson, the chairman of the committee, and which covered nine months only, as the end of the financial year is now December and not March, remarked that "the four private wards had proved of increasing value and importance." From March to December 46 patients were received in them, and their fees realised over £150. The ordinary income has increased to £5,558, and the ordinary expenditure amounted to £6,527, an increase also, but the hospital's building debt has been paid. The new nurses' home, the new theatre, and extension of the wards cost £10,852. The number of patients in the public wards was 980, and 24,263 persons were treated as out-patients. There is a deficit of £968, which remains to be paid.

**York County Hospital.**

The last year's work of the York County Hospital was summarised by the Dean of York, who presided at the annual meeting. He said: They would see that the number of in-patients had increased during the year, the average number of in-patients had increased, the number of out-patients had increased, and the attendance of out-patients had increased also. The income at the present time was increased, and the special appeal which had been made in order that they might bring themselves into a solvent condition, had been wonderfully answered, a balance of £200 being in hand. Another healthy feature was the Workpeople's Hospital Fund, which had been raised to £1,207, the highest since the fund was instituted ten years ago. Mr. Riley Smith was thanked heartily for his share in raising the money appealed for. Major Dent, one of the hospital's trustees, deprecated strongly the system of running up debts for three or four years, and then endeavouring to save the situation by appeal. Major Dent added: "The trustees are very loth to take steps to close a ward, but if 107 patients cannot be treated without £1,000 of debt, the only thing to do was to treat fewer patients."

ARMY MEDICAL LIBRARY

A notable statement on hospital policy by Sir Edward Wood gave particular interest to the recent annual meeting of the Leicester Infirmary. He said that he anticipated the time when a Central Hospital Board could be established for the county and district after the manner of King Edward's Hospital Fund. He considered it would be for the welfare of all the institutions that they should be under one Central Board, while managed by local committees. At present there was a clashing of interests. The spirit that was now growing in favour of voluntary hospital work was so great that he felt the hope he had expressed had only to become widely known to bring about very happy results. Certainly, as Sir Edward Wood remarked also, the Leicester Infirmary has received a larger share of public sympathy than ever, and a glance at last year's work shows that this has been earned. The in-patients numbered 3,358 and the out-patients 12,856, and 1,405 patients were sent to convalescent homes by the Saturday Hospital Society and the Infirmary. The annual expenditure was £21,071 and the income £21,531, leaving a surplus of £460. The invested capital was £70,000, against £35,000 nine years ago. The expenditure for the year is estimated at £22,000, and the authorities hope to be freed from anxiety as to the raising of this sum. The Mayor of Leicester (Alderman Vincent) presided.

**Sir Edward Wood's Forecast.**

Under the distinguished patronage of H.R.H. the Duke of Connaught, the committee of the Royal Southern Hospital, Liverpool, has inaugurated a "Half Million Shilling Fund." The objects of the appeal are (1) to relieve the hospital of a debt of £8,000, (2) to avert re-building by modernising the interior, and (3) to provide additional accommodation for patients and staff. It is nearly forty years since the present building was erected, and with the advances in medical and surgical science and hospital construction, in the interval, a large sum of money is now required to restore the buildings and to make them hygienic. The wards and corridors require re-flooring, the ventilation and heating needs revision, the sanitary arrangements are almost obsolete from a modern point of view, and the accommodation for resident medical, nursing and clerical staffs is inadequate. The hospital also needs observation and isolation wards, clinical laboratories, and re-modelled receiving and emergency dressing-rooms. With these wants facing the committee, coupled with the large debt, it is hoped that the appeal will receive a ready response. The public is bound to recognise the fact that buildings which are forty years old, instead of being regarded as barely seasoned, as they would have been in the fifteenth century, in the twentieth are antiquated in the extreme.

**Southern Hospital, Liverpool.**

The annual meeting of the Governors of the British Lying-in-Hospital, Endell Street, was held at the hospital on Tuesday last week. In moving the adoption of the report and accounts for the year 1910, the chairman, Mr. Charles E. Farmer, called attention to the fact that, notwithstanding every effort was made to keep down expenditure, the amount of the deficit for the year was £465, the expenditure being £151 less than in 1909. He considered it extraordinary that legacies were scarcely ever left to the British Lying-in-Hospital. It was hoped, however, that the forms of bequest sent out with the annual report might help in this direction. The chairman regretted that, in order to meet accumulated liabilities, it had been necessary to sell invested funds to the extent of £1,755. This, of course, was accounted for in the balance, though the actual sale was not recorded there. He hoped, however, that, with the continued kind assistance of the Ladies' Committee and their friends, to whom the hospital was so heavily indebted for the deep interest taken by them in its welfare, in course of time income and expenditure might more nearly balance. Mr. Farmer added that he had served on the Board of Management for some twenty-seven years now, and never, with the exception of sales in connection with building of the nurses' home, had he, until the last few years, had the experience of realising invested funds to meet the expenses of the charity. The chairman directed attention to the fact that the subscription list, the backbone, as it were, of revenue, showed an increase, which was very satisfactory, but he would like to see a still further advance. In speaking of the report of the Samaritan Fund, the chairman again called attention to the good work of the ladies, and said how indebted he felt to them for all they had done and were doing, and how pleased he was to see some present at the meeting. Pound Day, organised by the ladies, was last year a brilliant success, £74 in money and 17 cwt. in groceries, etc., having been contributed. During the year 470 in-patients and 610 out-patients were treated. Arrangements have been made for paying-patients, and those who live in lodgings and who cannot afford the cost of a nurse will find them invaluable for their comfort and their purses. It is to be hoped that this year a fair share of legacies may be received.

**British Lying-in-Hospital Meeting.**

As we have stated already, Worthing's memorial to King Edward is to take the very practical form of a new out-patients' department to the hospital. For some time past the existing out-patients' department has been quite inadequate to cope with the steadily increasing demands upon it, and a requisition, numerously and influentially signed, was sent to the Mayor (Alderman E. C. Patching, J.P.) requesting him to call a public meeting on the subject. His Worship took up the matter with the greatest keenness, and, in addition to commending the scheme, headed the list of subscribers with a donation of twenty guineas. Mr. H. R. P. Wyatt, J.P., Chairman of the Committee of Management, spoke eloquently in favour of the proposal, and subscribed a similar sum. So far some £400 has been subscribed, and the Mayor is issuing an appeal to meet the cost of the new wing, which has already been begun, and will cost some £3,000. It is hoped that the building will be opened in September. The plans provide for a large waiting hall, two consulting rooms, surgery and dispensary, and two dressing-rooms, with drug-store on the ground floor; five bedrooms for the nurses, with bathrooms, etc., on the first floor; and the new building will be connected with the hospital by means of a covered way. The building will be both useful and ornamental, and it is hoped that the cost will be defrayed by the time that it is ready for use.

**Building at Worthing Hospital.**

The annual meeting of the Governors of the Great Northern Central Hospital was held last week, Mr. H. J. Tennant, M.P., deputy-chairman, presiding. The report stated that the in-patients numbered 2,051, while the new out-patients were 24,347. The debt to the bankers has been reduced to £2,800, including a debt of £1,550 on the building fund, which it is desired to pay off at the earliest possible moment. A new home for the nursing staff was stated by Mr. H. J. Tennant to be the next aim of the committee, but this cannot be provided till not only has a capital sum been raised, but also a number of new annual subscribers been obtained to replace the continual falling off under this heading. Votes of thanks to the staff concluded the meeting.

**Great Northern Cent Hospital.**

